# Is the Eruption of Teeth Caused by Blood-Pressure?

**Published:** 1903-10

**Authors:** 


### Editorial.

### IS THE ERUPTION OF TEETH CAUSED BY BLOOD-PRESSURE?

In this journal for June was published an interesting paper, read by Thomas E. Constant, M.R.C.S., of England, before the Section on Stomatology, American Medical Association, at New Orleans, entitled "The Dental Pulp viewed without the Microscope." He presented in this paper a very ingenious and novel theory that we are "justified in assuming that the blood-pressure exerted in the vascular tissue, which lies between a developing tooth and its bony surroundings, is the active mechanical factor in the process known as the eruption of teeth." In the September number will be found the discussion upon the paper, equally interesting, as it gives the views of some of the best scientific minds in the dental profession.

The subject is worthy of further elaboration, not with the expectation of shedding light upon an abstruse physiological fact, but, possibly, to enlarge the sphere of criticism of the explanation so lucidly given. The subject has not, through the paper or discussion which followed, passed beyond the limitation of theory, for until we are in possession of more facts of observation it cannot be considered, by any means, as settled.

There is no one subject connected with the development of teeth that has puzzled the thinking mind more than this. Teeth make their way to the surface in an undeveloped state, and equally are extruded from their sockets through the loss of antagonists. The two processes, while seemingly the same, are evidently the result of diverse action, and cannot be confounded with each other. Constant contends that the latter condition is equally the result of blood-pressure, for he says, "The vascularity of this membrane (peridental) still endows it, however, with an extrusive tendency, which is in itself sufficient to account for the gradual elongation of unopposed teeth, and is probably the chief means by which the proper occlusion of opposing teeth is maintained."

The difficulty of accepting this conclusion lies in the fact that the elongated tooth cannot be regarded as being in a pathological condition, for it is practically undisturbed in its socket, and, being thus normally quiescent, there can be nothing to produce a hyperæmic state in the peridental membrane. With its normality unchanged, there could not possibly be an increased blood-pressure to force it out of its original position. The apt illustration, given long ago by Dr. Pierce, seems to be more nearly the true explanation,—that the tooth is forced out similarly to the bung in the barrel by a sudden blow on the staves adjoining. The tooth, no longer having the force of the occluding tooth, is left to the action of the surrounding alveolus, which, increasingly developing through loss of external pressure, produces a constant force upon the root and mechanically, through additions at the extremity and laterally, sends the tooth forward. This explanation is based on the fact that the formation of bone follows the gradual elongation.

The same reasoning will not, however, apply to the eruption of teeth during development. Another force comes in here,—the force of growth. Constant objects to this idea, for he says, "Since the forming root is never in actual contact with its bony surroundings, it must necessarily be against the vascular material in which it is embedded. Now, this tissue appears post mortem of far too jelly-like a consistence to oppose any effective resistance by virtue of its own structure, and yet such resistance there must be, or the tissue would be obliterated. Whence, then, are its resisting properties derived? Necessarily from the blood-pressure." This does not appear to the writer as conclusive. It is true that as growth proceeds vascularity is increased, and in that sense his view may be regarded as correct, but because of that increased vascularity it does not necessarily follow that the pulp is of "too jelly-like a consistence" to produce a force sufficient to cause the tooth to advance towards the surface.

The force of growth is apparently underrated by this writer and by some of those who enthusiastically adopted his view. The tissue of the developing pulp is ample, notwithstanding its jelly-like character, to produce a resistance that will send the tooth forward without recourse to the growth of the hard tissues which surround it. There are too many illustrations of this to require more than the mere statement, but the writer would call to mind the growth of a vascular tumor, not uncommon in the mouth, and where wide separation is made by its forcing the teeth widely apart. This is within the experience of almost every practitioner, and presents a greater force than would possibly be required to move a single tooth towards the gum. The vascularity of this tumor may be used as an argument in favor of blood-pressure, but if so, the greater including the less, the normal amount of blood in the developing tooth is quite equal to the lessened force demanded.

The development of plant-life presents similar equally interesting phenomena. The plant germ bursts its investure and gradually advances towards the surface of the earth. Is this progress due to sap pressure? It will hardly be contended that this is the force that moves the plant through its natural environment, and yet, if the blood-pressure be the explanation of the eruption of the tooth in the animal, are we not forced to a similar conclusion when considering the eruption of the vegetable?

It is scarcely necessary to call attention to the force manifested in vegetable growth. All are familiar with rock rent in twain by vegetable fibres originally of the most delicate character. In fact, this growth illustrates very fully, it seems to the writer, the possibility of a force that sufficiently corroborates the idea that the pulp is capable of exerting a power quite equal to the demands made for tooth eruption. If an illustration be needed to enforce this view, attention is called to the physiological process of dentition. It is claimed by some that this is not a pathological process, and while this is true, the results are frequently pathological. When reflexes occur from this cause, what must be the conclusion? Simply, that growth has proceeded faster than absorption of the surrounding tissues and that, therefore, there is pressure of the developing pulp upon the superimposed hard bony envelope, and producing, through that pressure, severe systemic disturbance. If the pulp was of a too jelly-like substance to produce resistance in growth this could not possibly result, and we would have a continuance of normal conditions notwithstanding the pressure exerted. It may possibly be true that the pressure produces an increased blood-supply; in fact, it must result in hyperæmia, and, so far, the essayist's position has a certain modified support, but the condition is abnormal, indeed pathological, and bears no relation to the physiological process of eruption in dentition. This must go on to completion without a blood-supply other than normal.

While the question is one of great interest, neither the valuable paper of our English colleague nor the discussion has cleared this complex subject of its difficulties, nor is it pretended that this article will add materially to general enlightenment. From whatever side the subject is viewed, problems, not as yet solved, present themselves, and it is more than probable that these will continue to remain in the domain of theory. We must, however, gladly welcome every new thought in this direction, and Dr. Constant's explanation must be regarded as one of the most valuable contributions to a very difficult subject.

